# Ovarian granulosa cell tumors: a retrospective study of 27 cases and a review of the literature

**DOI:** 10.1186/1477-7819-11-142

**Published:** 2013-06-18

**Authors:** Sakina Sekkate, Mouna Kairouani, Badr Serji, Adnane Tazi, Hind Mrabti, Saber Boutayeb, Hassan Errihani

**Affiliations:** 1 Department of Clinical Oncology, National Institute of Oncology, Rabat, Morocco; 2 Department A of surgery, Ibn Sina hospital, Rabat, Morocco; 3 Epidemiology Unit, National Institute of Oncology, Rabat, Morocco

**Keywords:** Granulosa cell tumors, Outcomes, Ovary cancer

## Abstract

**Background:**

Granulosa tumors were described for the first time in 1855 by Rokitansky. These tumors are malignancies with a relatively favorable prognosis. They are characterized by a prolonged natural history and a tendency to late recurrences. The aim of this study is to investigate the epidemiological and pathological characteristics of granulosa cell tumors and to investigate the prognosis factor for recurrences.

**Methods:**

The clinical data of patients who were treated in the period from January 2003 to December 2010 at the National Institute of Oncology in Rabat, Morocco for adult granulosa cell tumors of the ovary were investigated retrospectively. Data for age, clinical manifestation, imaging, diagnosis and treatment of the patients were reviewed and analyzed. Post-operative histology was obtained for all patients.

**Results:**

Twenty-seven cases were retrieved. The median patient age was 53 years. The most common clinical manifestations at diagnosis were abdominal pain and vaginal bleeding. Mean tumor size was 14 cm.

The majority of patients had stage I (63%, n = 17), while (18,5%, n = 5) had stage III, (7.4%, n = 2) had stage IV, and (11%, n = 3) of patients had an unknown stage.

In the follow-up period (median = 63.44 months), five (18.51%) patients relapsed. The median time to relapse was 41.8 months, (range: 18 to 62 months).

**Conclusions:**

Granulosa cell tumor of the ovary is an uncommon neoplasm. The adult form progresses slowly and often is diagnosed in an early stage of disease. Surgery is indicated. A prolonged post-therapeutic follow-up is necessary because of the risk of recurrences, late and exceptional for the adult form.

## Background

Granulosa cell tumors are very rare ovarian malignancies; they represent 2 to 3% of all ovarian cancers and mainly occur within the adult population [1]. They arise from sex cord tumors and stroma. In comparison to epithelial ovarian cancers, they are characterized by a good prognosis [2]. There are two histological forms: an adult form (95%) [3] and a juvenile form (5%), which is characterized by occurrence at an early age, with more pronounced signs of malignancy and an increased risk of recurrence [3,4].

These tumors have a particular clinical, histological and evolutive profile, and may reoccur up to 40 years after diagnosis [5].

Complete surgical resection is the mainstay of treatment, particularly in the case of early stage patients. Surgery has to be combined with platinum-based chemotherapy for advanced stages [6].

The aim of this study is to report the epidemiologic, anatomo-clinical characteristics and to determine the prognostic factors of survival.

## Methods

### Clinical data

This is a retrospective study of patient data originally collected between January 2003 and December 2010. A total of 27 patients were diagnosed with granulosa cell tumors during that time period by the Department of Clinical Oncology, in the National Institute of Oncology based in Rabat, Morocco.

The recorded information includes, age, parity, menopausal status, symptoms, diameters of tumors, stage of disease, type of surgery, adjuvant treatment, survival in months, recurrence and mortality.

### Follow-up

Patients were followed up until November 2012.

### Statistical analyses

The statistical analyses were done using SPSS 10.0 software. Department of statistics, National Institute of Oncology, Rabat, Morocco.

In this analysis, clinical data is expressed in percentages.

## Consent and statement of ethical approval

As the treatment of each patient was decided by the medical staff of the centre, oral consent was obtained from the subjects and was approved by the institutional review boards of the National Institute of Oncology, Cancer Centre in Rabat. This study was approved by the institutional review boards of National Institute of Oncology, in Rabat.

## Results

### Clinical features

During the period from January 2003 through December 2010, 27 patients underwent surgery for the adult form of granulosa cell tumors. The mean age of the patients was 53 years.

For 70% of the patients, the tumor occurred between the fifth and seventh decades. The mean parity was 3.14, and 41% of the patients (n = 11) were menopausal.

A total of 59% of the patients (n = 16) presented abdominal pain at diagnosis and also presented with vaginal bleeding as follows: intermenstrual bleeding (37%, n = 10), postmenopausal bleeding (19%, n = 5).

Other symptoms included abdominal distension (44% n = 12) and amenorrhea (11%, n = 3).

The average tumor size was 14 cm (range: 7 to 30 cm).

A summary of patient characteristics is presented below in Table 1.

**Table 1:** Characteristics of the patients

**Characteristics (n = 27)**	**Number of patients (%)**
**Age, median (years)**	53
➢ Third decade of life	1 (4%)
➢ Fourth decade of life	3 (11%)
➢ Fifth decade of life	8 (30%)
➢ Sixth decade of life	5 (18%)
➢ Seventh decade of life	6 (22%)
➢ Eighth decade of life	4 (15%)
**Symptoms at diagnosis**	
➢ Abdominal pain	16 (59%)
➢ Abdominal distention	12 (44%)
➢ Postmenopausal bleeding	5 (19%)
➢ Intermenstrual bleeding	10 (37%)
➢ Amenorrhea	3 (11%)
**Tumor size** (mean size: 14 cm)	
➢ ≤10 cm	8 (30%)
➢ >10 cm	19 (70%)
**Stage**	
➢ I:	17 (63%)
Ia	12 (44%)
Ic	5 (19%)
➢ III	5 (18.5%)
IIIa	1 (4%)
IIIc	4 (15%)
➢ IV	2 (7.5%)
➢ Unknown	3 (11%)

### Treatment

Twenty-two patients (81%) underwent hysterectomy with bilateral salpingo-oophorectomy with optimal resection (R0), omentectomy, +/− lymphadenectomy and multiple biopsy. Three patients (11%) had unilateral oophorectomy, and two patients (7.4%) had debulking surgery.

Thirteen patients had endometrial biopsies. The results were as follows: four were negative, six were hyperplasic, and three were atrophic.

Eight patients received adjuvant treatment (four: bleomycin etoposide, cisplatin (BEP); one: bleomycin vepeside cisplatin (BVP); one: endoxan cisplatin; one: paclitaxel cisplatin; one: tamoxifene), and two patients received chemotherapy for metastatic disease (one: BVP; one: BEP).

### Staging

The staging breakdown was as follows: stage I (63%, n = 17), stage III (18.5%, n = 5), and stage IV (7.5%, n = 2).

For the remaining 11% of patients (n = 3), the stage was unknown.

### Survival

**Figure 1:**
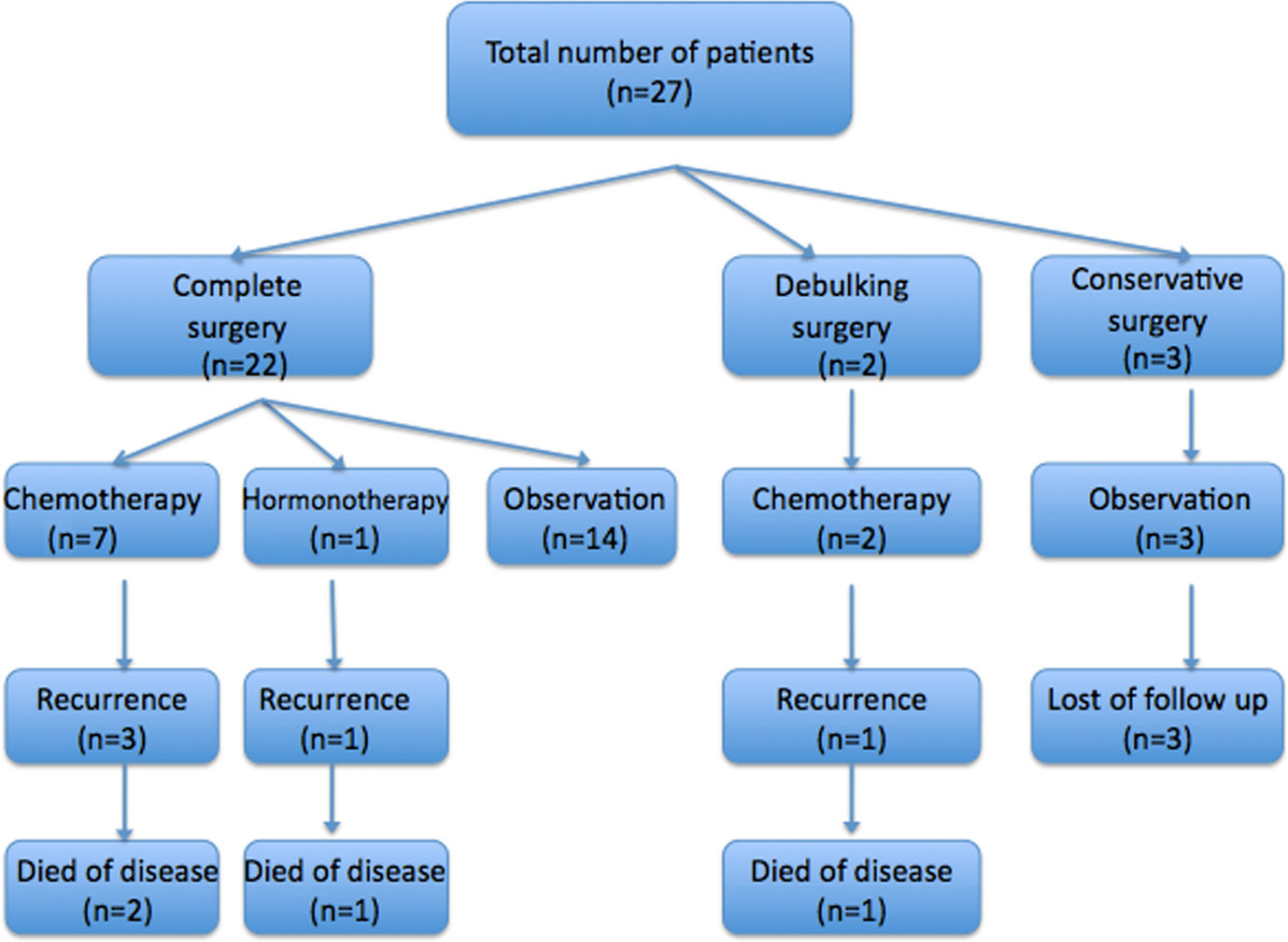
Management and outcomes of patients.

During the follow-up (median: 63.44 months), five patients (18.5%) relapsed, and four of those patients died of the disease (Figure 1).

Patient characteristics for those patients with recurrent disease are shown below in Table 2.

**Table 2:** Characteristics of patients with recurrent disease

**Case**	**Age (years)**	**Stage**	**Size of tumor (cm)**	**Primary treatment**	**Time to relapse (months)**	**Site of relapse**	**Treatment for relapse**
1	73	IIIc	20	Deb Sur + CMT	18	Pelvic	-
2	64	IIIa	20	Com Sur + CMT	55	Perit	Surgery
3	47	IV	15	Com Sur + CMT	62	Lung	CMT
4	76	Ia	10	Com Sur + Horm	38	Perit	-
5	41	IIIc	11	Com Sur + CMT	36	Perit	-

*Com Sur*, complete surgery, *CMT* chemotherapy, *Deb sur* debulking surgery, *Horm* hormonal therapy, *Perit* peritoneum.

The median time to relapse was 41.8 months, (range: 18 to 62 months).

**Figure 2:**
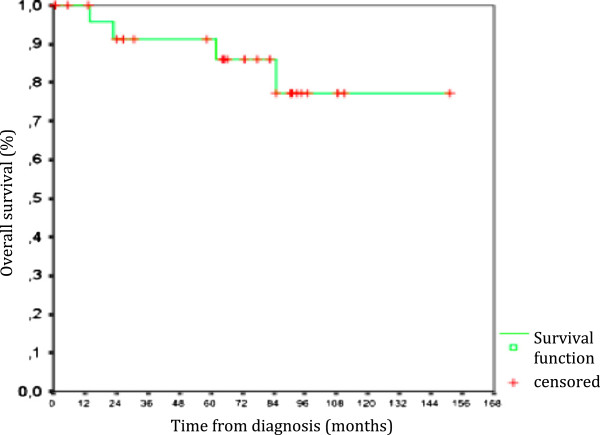
Overall survival at 5 years and 9 years for all patients.

The overall 5-year survival and 9-year survival rates for all stages were 91.3% and 77.3%, respectively (Figure 2).

**Figure 3:**
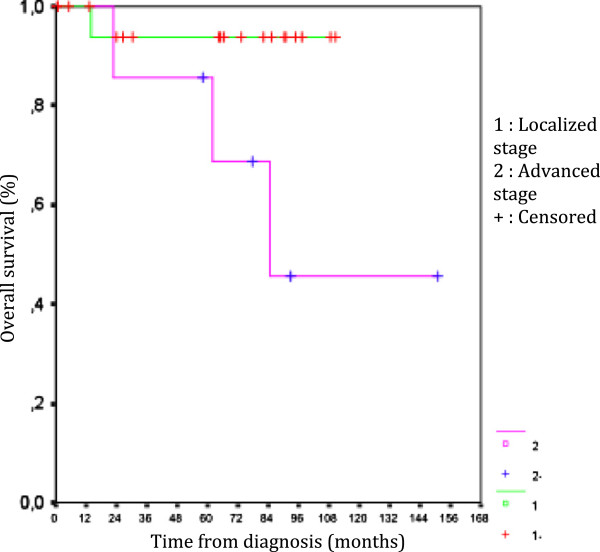
Overall survival by stage.

Following univariate Cox regression modeling, survival appears to be dependent on the stage, as it is much better in localized stages (*P* = 0.05) (Figure 3).

Other parameters considered in this study did not significant influence survival.

## Discussion

Granulosa cell tumors are very rare. They were described for the first time in 1855 by Rokitansky [7], who described them according to their appearance near the granulosa cells of ovarian follicles. They occur in the peri- and postmenopausal period with peak prevalence in patients aged 50 to 55 years. The other peak frequency corresponds to the prepubertal age [8,9].

The symptoms are various: abdominal pain (30 to 50%), abdominal distension related to mass effect and hormonal events (41%) such as irregular menstruation, intermenstrual bleeding, postmenopausal bleeding or amenorrhea [10].

However, for those cases in which the patient is asymptomatic, the clinical exam is very important [11].

Endocrine manifestations are noted in 66% of the patients. These manifestations are related to estrogen secretion of the tumor [12].

This explains why the granulosa cell tumors are frequently associated with endometrial hyperplasia (4 to 10%) or to endometrial adenocarcinoma (5 to 35%) [4,9,10].

Therefore, endometrial and cervical biopsies are essential to define the therapeutic strategy.

The juvenile form can be characterized by the presence of pseudopuberty (50% depending on the series) [13,14], and galactorrhea may complete the clinical presentation. The mechanism is not clearly established [9].

Radiologicaly speaking, the granulosa cell tumor presents as a solid component with multicystic appearance, with a median diameter of 12 cm (range: 1 to 30 cm) [15,16].

The imaging appearances of the two forms of granulosa cells tumors are similar.

The same clinical and radiological data were noted in the patients from our study.

The diagnosis is confirmed by histological analyses. The adult form includes five subtypes, among which the most common subtype - microfollicular - is characterized by Call-Exner bodies and cores “coffee bean” [17,18].

In the juvenile form, the architecture is often lobulated, Call-Exner bodies are rare, and the signs of luteinization are frequent [7].

The main immunohistochemical markers expressed by these cells are vimentin, CD 99 and alpha inhibin.

The serum tumor markers are estradiol, inhibin, and anti-Müllerian hormone. Cancer antigen 125 (CA-125) is not correlated to the tumor progression [18].

Kalfa *et al*. [19] identified a mutation *FOXL2* (transcription factor gene) in the majority of granulosa cell tumors, particularly in adult form. This *FOXL2* could be the next target for use in treatment.

Yoo *et al*. [20] also identified mutations of genes *Fas*, *FLIP* and *Bcl-2* related to alterations of apoptosis.

The principal differential diagnoses of granulosa cell tumors are: endometrioid carcinoma, stromal sarcoma, carcinoid tumors and adenocarcinoma [21].

Various factors determine the prognosis. The most important prognostic variable is the stage.

The survival rates at 5 years and 10 years were reported by Malmstrom *et al*. [2], 94% and 88%, respectively, for stage I, and decreasing to 44% for stage II and III.

Wu *et al*. [22] also reported their results about survival for 100 patients with granulosa cell tumors; survival rates at 5 years and 10 years were 98% and 96%, respectively, for stage I and were 70% and 60%, respectively, for stage II. The recurrence rate is also related to the stage [23].

The results of Ahyan’s study of 80 patients with granulosa cell tumors, revealed recurrence rates of 5.4%, 21% and 40% for stage I, stage II and stage III, respectively [24].

The prognostic value of stage was also noted in our study.

An age younger than 40 years is associated with a better prognosis, but the opinions differ [4,9].

In Ahyan’s trial, patients aged below 60 years had better mean time of survival (154.6 versus 89.2 months, *P* = 0.015) [24].

For most authors, larger tumor size is associated with a poor prognosis, particularly tumors that measured more than 10 cm [10,25].

Residual disease after surgery is also another prognosis factor. In Sehouli’s trial [26], the survival was lower for patients with postoperative residual disease.

The number of mitoses is also a recognized prognostic factor and there is an inverse relationship between survival and the number of mitoses [8,17].

Many studies, including the Schumer trial [27], proved that tumor rupture is also a prognosis factor.

Expressions of P53 mutations are common and may be associated with poor prognosis [28,29].

Ala Fossi *et al*. [30] noted that survival of patients with no mutations of P53 was 10 times higher than for patients with mutations.

Concerning inhibin, its value may be correlated to the tumor mass, with an increased level of inhibin in serum preceding clinical relapse [31,32].

The mainstay of treatments are complete surgery (hysterectomy, bilateral salpingoopherectomy) with staging for early stage and debulking surgery for advanced stage or recurrent disease [33].

There is no standard regimen concerning adjuvant treatment, but it is usually recommended for the adult form of granulosa cell tumors and for patients at high risk [2,23,27].

The most used chemotherapy regimen is a BVP (bleomycin, vinblastine, and cisplatin) or a BEP regimen, which substitutes etoposide for vinblastine [21].

The hormonal therapy based on megestrol and LHRH (luteinizing hormone-releasing hormone) agonists also lead to good responses, particularly for recurrent disease cases [34,35].

Rico *et al*. [36] demonstrated an increase in Mtor (mammalian target of rapamycin) deregulation by using a mouse model with granulosa cell tumors. So, targeting Mtor may be beneficial to women with granulosa cell tumors. More studies will be necessary.

For results of survival, the overall survival (approximately 90% at 5 years for early stage) is good, because most tumors are diagnosed early [16,22].

The evolution of adult granulosa cell tumors is slow and recurrences are rare and often delayed. These tumors can reoccur after a free interval of 6 to 23 years [10,21].

## Conclusions

Granulosa cell tumor of the ovary is an uncommon neoplasm. An important prognosis factor is stage at initial diagnosis. Due to the rarity of this disease, several prospective studies must be reported to establish a consensus.

## Abbreviations

BEP: Bleomycin etoposide cisplatin; BVP: Bleomycin vinblastin cisplatin; CA-125: Cancer antigen 125; LHRH: Luteinizing hormone-releasing hormone; MTOR: Mammalian target of rapamycin.

## Competing interests

The authors declare that they have no competing interests.

## Authors’ contributions

SS drafted the manuscript. MK contributed to the follow-up of patients. BS contributed to the conception and design of the manuscript. FE and SB helped with the literature research. AT contributed to the acquisition and analyses of data. HE approved the treatment and analyzed the literature data. All authors read and approved the final manuscript.
